# Molecular Detection of *Nosema* spp. in Honey in Bulgaria

**DOI:** 10.3390/vetsci9010010

**Published:** 2021-12-28

**Authors:** Delka Salkova, Rositsa Shumkova, Ralitsa Balkanska, Nadezhda Palova, Boyko Neov, Georgi Radoslavov, Peter Hristov

**Affiliations:** 1 Department of Experimental Parasitology, Institute of Experimental Morphology, Pathology and Anthropology with Museum, Bulgarian Academy of Sciences, 1113 Sofia, Bulgaria; dsalkova@abv.bg; 2 Research Centre of Stockbreeding and Agriculture, Agricultural Academy, 4700 Smolyan, Bulgaria; rositsa6z@abv.bg; 3 Department “Special Branches”, Institute of Animal Science, Agricultural Academy, 2230 Kostinbrod, Bulgaria; r.balkanska@gmail.com; 4 Scientific Center of Agriculture, Agricultural Academy, 8300 Sredets, Bulgaria; nadejda_palova@abv.bg; 5 Department of Animal Diversity and Resources, Institute of Biodiversity and Ecosystem Research, Bulgarian Academy of Sciences, 1113 Sofia, Bulgaria; boikoneov@gmail.com (B.N.); gradoslavov@gmail.com (G.R.)

**Keywords:** *Apis mellifera*, DNA analysis, health status, pathogens, molecular identification

## Abstract

Environmental DNA (eDNA) analysis is related to screening genetic material of various organisms in environmental samples. Honey represents a natural source of exogenous DNA, which allows for the detection of different honey bee pathogens and parasites. In the present study, we extracted DNA from 20 honey samples from different regions in Bulgaria and tested for the presence of DNA of the ectoparasitic mite *Varroa destructor,* as well as *Nosema apis* and *Nosema ceranae*. Only *Nosema ceranae* was detected, showing up in 30% of all samples, which confirms the widespread prevalence of this pathogen. All positive samples were found in plain regions of the country, while this pathogen was not detected in mountainous parts. None of the samples gave positive amplifications for the *Nosema apis* and Varroa mite. The obtained results from this study confirm previous observations that eDNA contained in honey is a potent source for effective biomonitoring of actual diseases in the honey bee.

## 1. Introduction

The Western honey bee (*Apis mellifera* Linnaeus, 1758) is a species with economic, agricultural, and environmental significance. Honey bees are known as the most effective pollinators because they pollinate various crops, fruits, and vegetables [1,2], thus supporting the existence of a large number of animal and plant species, including humans [3,4].

Honey bees are threatened by various pests and pathogens, some of which inflict heavy annual losses in the beekeeping sector. Among these threats, the ectoparasitic mite *Varroa destructor*, the fungal pathogens *Nosema apis* and *Nosema ceranae*, often acting simultaneously with some honey bee-associated viruses, are suspected to contribute considerably to honey bee colony losses [5,6,7,8]. A large number of methods and techniques have been developed for the identification of various honey bee pathogens [9,10,11]. One of the most widely used methods for the detection of a particular pathogen is the amplification of coding and/or noncoding DNA regions, followed by a sequence analysis of the amplified DNA fragment [12,13,14].

Most often forager bees and, less frequently, house bees, drones, or brood are used as a source of biological material for the identification of a particular pathogen. Alternatively, a large number of honey bee pathogens may be detected in honey bee products—usually, honey, propolis, and bee wax [14,15,16,17,18].

The term environmental DNA (eDNA) is most commonly used for the genetic material isolated directly from environmental samples (soil, sediment, water, etc.) without any obvious signs of biological material from external source eDNA [19]. This approach is a credible strategy for correct identification of cryptic species or juvenile life stages, elusive, or invasive organisms, including many pathogens that might be difficult to sample and/or identify [20,21]. Environmental DNA has been obtained from ancient and modern animals and plants samples, thus identifying not only single organisms but whole communities as well. This technique has recently been implemented successfully in the beekeeping sector. For example, the analysis of eDNA in pollen and some bee products (wax, propolis, and honey) has been used to detect the level of environmental contaminants and pollutants and to identify their origin [22,23]. Additionally, some bee products and, in particular, the analysis of eDNA in honey (i.e., derived from pollen) can be used for the evaluation of honey bee foraging behavior and the identification of the botanical composition of pollen [24,25]. Moreover, since plant-sucking insects produce honeydew, which honey bees regularly collect, these Hemiptera species can be identified on the basis of DNA traces in honey [26]. The detection of fungal and bacterial DNA in honey can be used for forensic and food safety analyses [27]. The following approaches have been adopted in the analysis of DNA contained in honey: (i) use of specific PCR primers to identify a particular species; (ii) use of universal primers for barcoding—the mitochondrial genes that are the most commonly used in bacteria are the small subunit of 16S DNA gene and cytochrome c oxidase subunit I gene (*cox1*) [17,28,29]; (iii) use of metabarcoding in combination with next generation sequencing (NGS) to allow determining the taxonomic composition of pooled honey eDNA [30,31]. As for the identification of single pathogens in honey, this approach is a non-invasive and relatively fast way to assess the health status of bee colonies as well as to identify some diseases without clinical symptoms [28,29].

Considering the above, we have conducted, for the first time in Bulgaria, a purposeful study on the detection of some of the most widely distributed pathogens of honey bees by using honey of different botanical and geographical origin as a source of eDNA. We have decided to use this relatively new approach due to the fact that it represents an alternative, yet effective, strategy for establishing the epidemiological status and monitoring of various honey bee diseases.

## 2. Materials and Methods

### 2.1. Honey Samples

**Figure 1:**
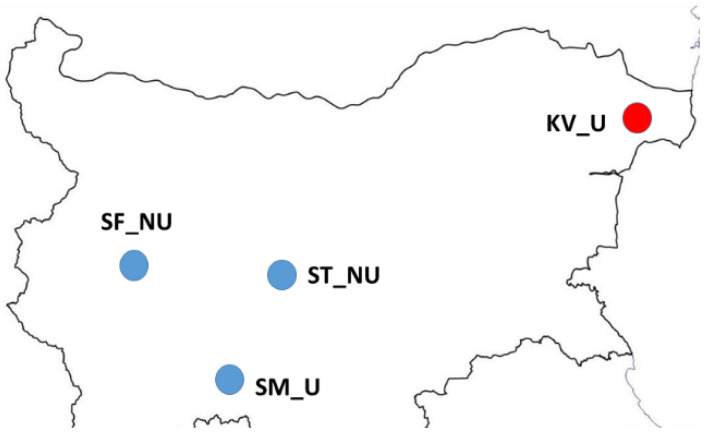
Map showing the locations of 20 honey samples in Bulgaria. Sample names indicate the region in Bulgaria (SF, Sofia City Province; SM, Smolyan; ST, Strelcha; KV, Kavarna). Habitat indicator (U, urban area; NU, non-urban area). Blue circles—polyfloral honey; Red circle—monofloral honey.

A total of 20 honey samples, originating from four different regions in Bulgaria (Northeastern, Western, Central and South Bulgaria) referred to as KV, ST, SF, and SM, respectively, were produced and collected in the summer of 2020 (Figure 1). In each geographical location, five honey samples were taken from single hives in each apiary. There was no bias concerning the obtained honey samples. The first two regions comprise generally flat plains, while the last two regions are situated in the mountainous parts of the country (Vitosha and the Rhodope Mountains, respectively). The honey samples were collected from hives within the same apiary. According to all producers, the apiaries had no visible clinical symptoms of any diseases.

According to the Bulgarian Food Safety Agency (BFSA), the results of an epizootiological survey in different areas of the country in 2020 showed that bee colonies are most often affected by varroasis and nosemosis, while in the previous year varroasis predominated as a cause of mortality in bee colonies. As far as the regions that we have studied are concerned, from 1 January to 30 April 2020, a total of 243 samples were tested; 23 of them were positive for varroasis, and 56—for nosemosis. The predominant part of the studied samples showed the presence of *V. destructor* and *N. ceranae* in Central and Northeastern Bulgaria. Moreover, in the regions observed in 2020, 37% of the samples showed a mixed invasion of varroasis and nosemosis, 33.3%—only varroasis, and 14.8%—only nosemosis. These data gave us reason to focus our research particularly on the molecular detection of *V. destructor* and *N. ceranae* in the honey samples.

### 2.2. DNA Extraction for Identification of eDNA from V. destructor, N. apis, and N. ceranae in Honey Samples, Using CTAB Protocol with a Spin Column

Honey is mainly composed of carbohydrates, which comprise about 95% of honey’s dry weight. It has been noted that the high concentration of sugars reduces the amount of DNA yields and inhibits PCR reactions [32]. Therefore, as an initial step in the process of DNA isolation, it is necessary to reduce the sugar content. Briefly, for each honey sample, a total of 50 g of honey was divided into four 50 mL conical centrifuge tubes (12.5 g for each tube). Subsequently, 37.5 mL ddH_2_O were added to each tube and incubated at 40 °C for 30 min to dissolve the honey. After the complete dissolution of the honey, the tubes were centrifuged for 30 min at 5000× *g* at room temperature, and the supernatant was discarded. The obtained pellet was re-suspended in 5 mL of ddH_2_O, and the content of the four tubes was pooled together, and then diluted in 50 mL ddH_2_O. These steps were repeated three times. After the last centrifugation and discard of the supernatant, the pellet was dissolved in 1 mL of an autoclaved CTAB DNA extraction buffer (2% (*w*/*v*) cetyltrimethylammonium bromide, containing 100 mM Tris-HCl, pH = 8, 20 mM EDTA, pH = 8, 1.4 M NaCl), transferred in a 1.5 mL tube. Subsequently, 18 μL of RNase A solution (10 mg/mL, Cat. no. EN0531, Thermo Fisher Scientific, Inc. Waltham, MA, USA) were added to each honey sample and incubated for 30 min at 65 °C. CTAB buffer is suitable for DNA isolating because the CTAB is a cationic surfactant, which is why it binds with lipids from the cell membrane and provokes cell lysis. Additionally, in cells with a high concentration of sugars (as with honey) CTAB binds to the polysaccharides when the salt concentration is high, thus removing polysaccharides from the solution. After this incubation step, 30 µL of proteinase K (20 mg/mL) were added in each tube, and all samples were further incubated at 56 °C for 90 min with gentle shaking. After the inactivation of proteinase K for 10 min at 70 °C, the samples were transferred to nucleospin columns to obtain a high yield of DNA. Total DNA was isolated with a GeneMATRIX Tissue Kit (Cat. no. E3550, EURx Ltd., Gdansk, Poland), according to the manufacturer’s instructions. The quantity and quality of the extracted eDNA were checked spectrophotometrically and by 1% agarose gel electrophoresis in 1X TAE buffer staining with SimpliSafe™ (Cat. no. E4600, EURx Ltd., Gdansk, Poland) under UV light. The DNA was stored at −20 °C until PCR amplifications.

### 2.3. PCR Analysis

PCR amplification was performed using the PCR primer sets presented in Table 1.

We decided to use two primer sets to detect the presence of *V. destructor* (for fragments of *coxI* and *cytb* genes) in order to increase the possibility of identifying the ectoparasitic mite.

Before proceeding to PCR identification of the presence of some pathogens and parasites, we initially checked for possible amplification of eDNA from honey bees in honey samples. According to Ruttner’s morphometric analysis [37], *A. m. macedonica* exists as a native honey bee in Bulgaria. This honey bee subspecies belongs to the C evolutionary lineage, which is why we performed amplification of the mitochondrial *coxI-coxII* intergenic region according to the recommendation by Utzeri et al. [33]

The PCR analysis used one primer set for each reaction (single PCR analysis). The PCR analysis was carried out using a Little Genius thermocycler (BIOER Technology Co., Ltd., Hangzhou, China) in a total volume of 50 μL. The PCR mixture contained 25 µL of NZYTaq 2× Colorless Master Mix (Cat. No. MB04002, Nzytech, Lisboa, Portugal) and 0.4 µM of each specific primer (FOR/REV). The PCR conditions were as follows: initial denaturation at 94 °C for 5 min; 35 cycles (denaturation at 94 °C for 30 s; primer annealing at 56 °C for 30 s; extension at 72 °C for 1 min), and final extension at 72 °C for 10 min. The PCR products were visualized by 1% agarose gel staining with SimpliSafe™ (Cat. no. E4600, EURx Ltd., Gdansk, Poland) under UV light. The fragment size was determined using Gene-Ruler™ 100 bp Ladder Plus (Cat. No. SM0323, Thermo Fisher Scientific Inc., Waltham, MA, USA). When there was no visible amplification or smears on the gel after the first PCR reaction, 5 µL were taken from the reaction mixture and were used as the DNA template for a second PCR reaction. This approach is a standardized procedure to suppress the formation of artificial chimeras during PCR amplification [38]. A second PCR reaction was not initiated when there was visible amplification after the first reaction. The successfully amplified products were purified by a PCR purification kit (Gene Matrix, PCR clean-up kit, EURx, Poland) and sequenced in both directions by a PlateSeq kit (Eurofins Genomics Ebersberg, Gdansk, Germany).

### 2.4. Sequence and Phylogenetic Analysis

All five obtained DNA sequences were manually edited and aligned in MEGA v7.0.20 [39], using the MUSCLE algorithm [40]. BLASTN (http://www.ncbi.nlm.nih.gov/BLAST/ (accessed on 15 October 2021)) was performed to compare the similarity and validity of the obtained sequences [41]. After the alignment, all five obtained sequences of *N. ceranae* showed complete homology. Therefore, only one of them was deposited in GenBank—under accession number MG657260.

In order to evaluate any geographic linkage, the obtained sequence of *N. ceranae* from Bulgaria was aligned with 18 other isolates from different geographical locations, using the reference sequence NW_020169312, a part of ASM98816v1 *Nosema ceranae* genome assembly [42]. Sequences were analyzed by polymorphic single-nucleotide polymorphism position.

## 3. Results

Environmental DNA extracted from all honey samples was successfully amplified with respect to the mitochondrial *coxI–coxII* intergenic region of *Apis mellifera*, obtaining fragments of the expected size. Thus, DNA was successfully isolated from all samples, and PCR amplification was not inhibited by any contaminants in the isolation process. Therefore, eDNA isolated from all honey samples was suitable for the molecular detection of three pathogens in a single PCR analysis.

When using the two pairs of primers for *V. destructor*, we found no amplification of the mitochondrial genes *coxI* and *cytb*, even after a second PCR. Apparently, the excessive size of the expected fragments in the presence of the highly degraded eDNA does not allow successful amplification. In contrast to the results regarding the ectoparasitic mite, eDNA in the honey samples was successfully amplified with respect to the fungal pathogen *N. ceranae*. We detected this pathogen only in plain regions—the KV honey samples (20%, 4/20) as well as in the ST samples (10%, 2/20). In the studied mountainous regions (SF and SM), the presence of *N. ceranae* was not established.

The results from the sequence analysis of the 16S rDNA from Bulgaria and 18 other sequences available in GenBank (https://www.ncbi.nlm.nih.gov/genbank/ (accessed on 25 October 2021) revealed that the newly obtained sequence from Bulgaria is practically identical to the sequences from Morocco, Lebanon, Egypt, Turkey, Argentina, Switzerland, Germany, France, and Japan, when compared with the reference sequence (GenBank Acc. no. NW 020169312) (Table 2). Our findings also show that it is difficult to find any geographical relationship between the different haplotypes. Moreover, the sequence obtained from a single isolate showed different nucleotide polymorphism from those obtained from distinct isolates in the same country (GenBank Acc. no. LC493173, LC510233 and LC510243 from Japan) (Table 2). Conversely, there are isolates with identical haplotypes in samples of very different geographical origin (GenBank Acc. no. LC510243 from Japan and GenBank Acc. no. HM581509 from Mexico).

In contrast to *N. ceranae*, the other fungal pathogen, *N. apis*, did not show amplification in any of the tested samples.

## 4. Discussion

Honey has always been an attractive and relevant subject of study, not only because of its nutritional value and content of micro and macronutrients, but also because of its widespread use as an alternative therapy (apitherapy) in medicine [43,44]. However, there are other biological molecules in the composition of honey, which have been used and studied relatively rarely, so far. Undoubtedly one of them, which has recently aroused considerable research interest in various scientific aspects, is eDNA [45,46].

In this study, we used conventional PCR analyses for molecular detection of some of the most widely distributed honey bee pathogens in honey samples. In this pioneering study for Bulgaria, we tried to use the non-invasive, relatively fast and innovative approach for DNA isolation in honey samples in order to evaluate and monitor bees’ health status. Honey bee DNA is always present in honey and can be used as a positive control even for the presence of traces of eDNA [33]. Therefore, the first step in this study was to detect the presence of bee eDNA and to assess its quality and quantity. This is a necessary condition that allows for the search and amplification of eDNA from other biological objects. After receiving the amplification of the mitochondrial *coxI-coxII* intergenic region in the subspecies *A. m. macedonica* in all tested samples, we proceeded with analyses for molecular detection of some bee pathogens.

As anticipated, no successful application was obtained for the ectoparasitic mite *V. destructor*. Apparently, the size of the expected fragments when using the two pairs of primers for the two mitochondrial genes *coxI* and *cytb* (929 bp and 985 bp, respectively) is too large to allow the application of the highly fragmented DNA in honey samples, as confirmed by previous studies [17,33]. The possibility of a negative result due to the absence of *V. destructor* DNA is extremely low.

It is also worth mentioning that a reason for the unsuccessful identification of the *V. destructor* might be not only the large size of the expected PCR fragments for *coxI* and *cytb* genes, but also the origin of the honey samples provided for analysis. Most of the studies in this direction use mixed honey from several hives in an apiary, or commercial honey, which largely resembles the mixed honey provided by beekeepers for commercial purposes [14,17,33]. This approach significantly increases the possibility of detecting a pathogen in honey samples.

Another limitation associated with the absence of residual mite DNA in honey may be the life cycle of this honey bee parasite. It is well known that the adult mites usually live and feed under the abdominal plates of adult bees primarily on the underside of the metasoma region [47]. At this location, the probability of *V. destructor* getting into the honey cells is very low.

Another pathogen that we were unable to identify in any of the analyzed samples was *N. apis*. This is not very surprising, given the reduction in the prevalence of this fungal pathogen and its replacement with *N. ceranae* in many geographical regions [48,49]. Our results confirm this trend, as *N. ceranae* was detected in 25% of the analyzed honey samples, only in the plain regions in the country (ST and KV).

Several explanations for these observations are possible. First, processes of increasing urbanization, observed in many countries, create favorable conditions for the spread of various pathogens in honey bees [50,51]. In such sites, contact between humans and animals, domestic and wild, is inevitable, which facilitates the dissemination of various pathogens [52]. The effects of increasing urbanization have been found to be additive rather than interactive, i.e., urbanization imposes changes not only on managed honey bee colonies but on feral populations with respect to their pathogens [53]. What is worrying is the fact that many of the beekeeping practices used and applied have also drastically changed the intensity of the invasion of various pathogens in honey bees [54,55]. In this regard, widespread prevalence of *N. ceranae* in poorly managed bee colonies has been found [56,57]. Our research has confirmed these observations, as we have found only infestation with *N. ceranae* in apiaries located in the KV honey samples (20% infestation level) and the ST honey samples (10% infestation level), but not in the mountainous parts in the country (the Vitosha Mountain—SF honey samples and the Rhodope Mountains—SM honey samples). The negative results obtained in the mountainous parts in the country could be due to the failure of amplification of the tested primer pairs, since we have not used primers for *N. ceranae* identification already tested by other authors [17,57]. Several PCR conditions were tested, with the same negative results for the presence of *N. ceranae* in SF and SM honey samples. The negative results for *N. ceranae* in the mountainous parts of the country could be due to for the presence of different strains across samples, having mutations in the primer regions that could affect the amplification efficiency. It will be interesting to further explore this issue.

The predominance of this pathogen only in the plains of the country could also be geographically determined. The mountainous regions are geographically isolated, and the opportunities for movement of people, goods, and livestock there are much more limited than in the plains. Moreover, exposure to insecticides in field conditions is much larger than in mountainous regions, which can increase colony susceptibility to various pathogens [58]. Another reason related to the establishment of *N. ceranae* only in the plains of the country may be associated with the fact that these regions provided a diet composed of pollen from diverse plants. In contrast, in the mountainous parts of the country the diet most often consists of pollen from a single plant species. As is well-known, the plains provide much greater opportunity for diverse honey vegetation than the mountainous regions (providing low-nutritional quality), which leads to a higher degree of infection [59].

Environmental conditions also affect *N. ceranae* dissemination and infestation rate, as it has been found that these microsporidia develop favorably in warmer climates [48,60]. In Bulgaria, the development of bee colonies in mountainous areas, e.g., the Vitosha Mountain (SF samples) and the Rhodope Mountains (SM samples), starts usually in May–June, i.e., about two months later than on the plains, which also contributes to the wider spread of *N. ceranae* in the plains.

The sequence alignment of *N. ceranae* isolate from Bulgaria along with other sequences available in the GenBank database (http://www.ncbi.nlm.nih.gov/ (accessed on 28 October 2021) has not revealed any differences among them, except for the single nucleotide polymorphism. These haplotypes are not specific in terms of geographical origin, so it is difficult to determine which isolate is specific for a given area, as well as to look for possible dependence regarding the spread of nosemosis worldwide. This is not surprising, given that our phylogenetic analysis of rDNA has not shown significant differences between sequences [61].

The present study on the use of eDNA analysis of honey samples for the molecular identification of some parasites and pathogens in bees has been, to our knowledge, the first research applying this innovative approach in Bulgaria. Although only one pathogen has been detected (*N. ceranae*), the obtained results (albeit preliminary) provide possibilities for the use of eDNA in monitoring the health status of bee colonies, the epidemiology, and the spread of some of the most common bee diseases.

## Figures and Tables

**Table 1:** Information on PCR primers used in this study to amplify eDNA from the honey samples.

Species	Primer Name	Primer Sequences (5′-3′)	Size in bp	Amplified Region	Reference
*Apis mellifera*	AM_Forward AM_Reverse	GGCAGAATAAGTGCATTG TTAATATGAATTAAGTGGGG	C lineage 85	mtDNA *coxI*-*coxII*	[33]
*Varroa destructor*	10KbCOIF1 6,5KbCOIR	CTTGTAATCATAAGGATATTGGAAC AATACCAGTGGGAACCGC	929	*coxI*	[34,35]
*Varroa destructor*	10KbCytbF-1 10KbCytbPRIM	GCAGCTTTAGTGGATTTACCTAC CTACAGGACACGATCCCAAG	985	*cytb*	[34,35]
*Nosema* *apis*	321APIS-FOR 321APIS-REV	GGGGGCATGTCTTTGACGTACTATGTA GGGGGGCGTTTAAAATGGAAACAACTATG	321	SSU 16S rDNA	[36]
*Nosema* *ceranae*	218MITOC-FOR 218MITOC-REV	CGGCGACGATGTGATATGAAAATATTAA CCCGGTCATTCTCAAACAAAAAACCG	218 or 219 ^a^	SSU 16S rDNA	[36]

^a^ The expected number of the amplified products in *N. ceranae* using the 218MITOC primers can be either 218 or 219, depending on the sequences for *N. ceranae* available in the GenBank database (http://www.ncbi.nlm.nih.gov/ (accessed on 25 October 2021)) [36].

**Table 2:** *N. ceranae* sequence variation among 19 isolates from different geographical locations. A 218-bp segment of the 16r DNA from 18 different geographical sites is aligned with the reference sequence NW_020169312 part of the ASM98816v1 *N. ceranae* genome assembly [42]. Variable nucleotides are indicated. Sequence positions above each column are given from the start of the reference sequence.

*N. ceranae* GenBank Acc. no.	6	6	6	6	6	6	6
	1	1	1	1	1	2	2
	8	8	8	8	9	0	0
	6	6	7	8	6	2	3
	2	7	7	4	3	9	5
NW 020169312 reference	A	A	A	A	A	T	A
MG657260 Bulgaria							
MW600361 Mexico				T			
MN649205 UK						A	
KC680638 Morocco							
KC680637 Lebanon							
MZ044978 Egypt							
MW396669 Turkey							
FJ227957 Argentina							
DQ673615 Switzerland							
DQ374656 Germany							
DQ374655 France							
LC493173 Japan					C		
LC510232 Japan NCS48		G					
LC510233 Japan NCS49	G						
HM581509 Mexico							G
LC510243 Japan NCS59							G
HM859897 Italy			G				
HM802210 Mexico			G				

## Data Availability

All data are available upon request.
